# Supplementary material for: Existence and features of the myodural bridge in Gentoo penguins: A morphological study
Source: PLoS One. 2021 Apr 8;16(4):e0244774. doi: 10.1371/journal.pone.0244774 (PMC8031436; doi:10.1371/journal.pone.0244774)
Supplement: S1 File — (DOC) [file pone.0244774.s001.doc]

**Staining methods**

**H&E staining**

1. Deparaffinize and rehydrate (routine)

2. Dye in hematoxylin (10 mins)

3. 1% hydrochloric acid alcohol (1 min)

4. Wash with running tap water

5. Wash with distilled water briefly

6. Dye in Eosin for 30 seconds

7. Perform regular dehydration

8. Clear in two changes of xylene (5 mins each)

9. Mount with neutral gum

**Masson trichrome staining**

1. Deparaffinize and rehydrate (routine)

2. Wash in distilled water.

3.   Rinse in running tap water for 5-10 minutes to remove yellow color.

4.   Stain in hematoxylin (10 minutes).

5. Rinse in running warm tap water (10 minutes)

6. Biebrich scarlet-acid fuchsin solution: 1% Biebrich scarlet 90 ml, 1% Acid fuchsin 10 ml, glacial acetic acid 1 ml. Dye for 10-15 minutes.

7. Wash in distilled water

8. Phosphomolybdic-phosphotungstic acid solution: 5% Phosphomolybdic acid 25 ml, 5% Phosphotungstic acid 25 ml, Differentiate in solution for 10-15 minutes

9. Aniline blue solution: Aniline blue 2.5 g, glacial acetic acid 2 ml, distilled water 100 ml. Transfer sections directly (without rinse) to aniline blue solution and stain for 5-10 minutes. Rinse briefly in distilled water and differentiate in 1% acetic acid solution for 2-5 minutes.

10. Wash in distilled water.

11. Regular dehydration (these step will wipe off Biebrich scarlet-acid fuchsin staining) and clear in xylene.

12. Mount with neutral gum

**Picrosirius Red (PRS) staining**

1. Deparaffinize and rehydrate (routine)

2 . Celestine blue solution：celestine B 1.25g, halotrichite 1.25g, distilled water 250ml. Boil to solve, filter after cooling down, add 30ml glycerin, and add concentrated sulfuric acid 0.5ml. Dye for 10 mins

3. Wash in distilled water (3 times, 1 minute each)

4. 0.1% sirius red solution (1X volume 1% sirius red in 9X volume saturated carbazotic acid, dye for 1.5 hours)

5. Wash with running tap water (5 mins)

6. Redye with hematoxylin (10 mins)

7 . Wash with running tap water (5 mins)

8. Perform regular dehydration

9. Clear in two changes of xylene (5 mins each)

10. Mount with neutral gum
