# Supplementary material for: Existence and features of the myodural bridge in Gentoo penguins: A morphological study
Source: PLoS One. 2021 Apr 8;16(4):e0244774. doi: 10.1371/journal.pone.0244774 (PMC8031436; doi:10.1371/journal.pone.0244774)
Supplement: Attachment — Submitted filename: Response to Reviewers.docx [file pone.0244774.s003.docx]

Dear editors:

We greatly appreciate both your help and that of the reviewer concerning the improvement of this paper. Thank you for the reviewers modifying sentences one by one patiently. We have read the given advice carefully, and we underwent considerable revisions on the article content (see the end of this mail). We have made some of the details of the manuscript easier to be understood, and more precise.

I hope you will give favorable consideration to the revised manuscript. I am looking forward to your earnest comments.

Sincerely yours

Academic editor

1. Please ensure that your manuscript meets PLOS ONE's style requirements, including those for file naming. The PLOS ONE style templates can be found at

https://journals.plos.org/plosone/s/file?id=wjVg/PLOSOne_formatting_sample_main_body.pdf and

https://journals.plos.org/plosone/s/file?id=ba62/PLOSOne_formatting_sample_title_authors_affiliations.pdf

Manuscripts have been ensured to meet PLoS ONE stylistic requirement, including document naming.

2. Please include both an updated Funding Statement and Competing Interests Statement in your cover letter. We will change the online submission form on your behalf.

**Funding Statement**

The funders had no role in study design, data collection and analysis, decision to publish, or preparation of the manuscript.

Funding: This research was supported by the National Natural Science Foundation of China (NSFC31871213 to HJS); and Department of Education of Liaoning Province (LZ2020048 to NZ). The funders had no role in study design, data collection and analysis, decision to publish, or preparation of the manuscript.

**Competing Interests Statement**

Haichang Ocean Park Holding Co., Ltd. is responsible for the breeding of Gentoo Penguin (*Pygoscelis Papua*) and provides the basic data related to the penguin specimens for this research group. Another part of their work is the study and popularization of science related to penguins. The authors have declared that no competing interests exist.

This does not alter our adherence to PLOS ONE policies on sharing data and materials.

Reviewer #1:

1.However, there are a number of aspects that need to be improved before it would be acceptable for publication. Most importantly, the discussion currently provides a very limited analysis of the specific anatomy in the target species and unique aspects might relate to specific aspects of species biology. A more thorough analysis of the comparative anatomy of the species included within the discussion and the relationship between this and the potential functional correlates is necessary.

Thanks for your advice, your advice of great value for our future study. At present, we only carry out a morphological study about the Gentoo penguin of "myodural bridge" to prove that "myodural bridge" universal existence and might have an impotant finction. For the next stage, related functional experiments will be carried out on the morphological abnormalities of different birds. Because "myodural bridge" in different kinds of vertebrates may have diffent morphologial characters due to diferent life style and living envionment. This will be an impotant study direction in our team.

2.There are numerous grammatical, typographical and formatting issues (some of which i have highlighted within the pdf document) that need to be addressed.

minor remarks:

Page 3

1. rephrase for clarity/style; e.g., The myodural bridge (MDB) is a...

Line 40: The myodural bridge (MDB) is an anatomical structure connecting a suboccipital muscle (RCPmi) to the cervical spinal dura mater (SDM) in humans was identified in the atlanto-occipital interspace by Hack et al. (1995) [1].

2. year required

Line 42: ....by Hack et al. (1995) [1].

3. delete,....With these in-depth studies, many researchers now speculate that the MDB may play a significant role in physiological functions.

Line 46: ....and the nuchae ligament (NL) [2-7]. ~~With these in-depth studies, many researchers now speculate that the MDB may play a significant role in physiological functions.~~ It has been proposed....

4. delte....s ....

Line 50: ....play an important role in modulating ....

5. change "rencent" to "also"

Line 51: According to also reports,

Page 4

1.italics not required here

Line 55: ....and sperm whales....

Line 56: ....Siamese crocodile and....

Line 57: ....Rock pigeons and....

2.insert binomial name

Line 64: Gentoo penguins *(Pygoscelis papua)*

3.fixed/preserved?

Line 72: ...."immobilized" was changed to "fixed and stored"

Page 5

1.insert relevant reference literature

Line 90: The Jiang Weizhong’s [31]

One literature has been added in this paper

[31]. Liu ZD, Fan QY, Qiu XX, Jiang WZ. 1998 . Morphological observation of bone remodeling in adult dogs. －Journal of the Fourth Military Medical University. (01), 108-109. doi:CNKI:SUN:DSJY.0.1998-01-045.

Page 7

1. was tightly adherent... (past-tense - inconsistent use of tense throughout)

Line 116: ....The dorsal atlanto-axial membrane was tightly adheret to....

2. insert binomial name in figure captions

Line 140: Gentoo penguins (Pygoscelis papua)

3. "membrane" and "was"

Line 132: membrane was

Page 11

1. Rephrase for clarity

Line 194: Therefore, what is unique about the penguin as a marine bird？

2.formatting issues

Line 200: the *Nephocaena phocaenoides*

Line 202: sperm whale

Line 204: and sperm whale

Line 206: Rock pigeons and

3.as described in previous research? references required here

Line 207: other birds (Rock pigeons and Gallus domesticus) [23, 24].

[23]. Chukwuemeka Samuel Okoye, Zheng N, Yu SB, Sui HJ. 2018. The myodural bridge in the common rock pigeon ( Columbia livia) : Morphology and possible physiological implications.－J Morphol. 279(10):1524-1531. doi: 10.1002/jmor.20890.

[24]. Dou YR, Zheng N, Gong J, Tang W, Chukwuemeka Samuel Okoye, Zhang Y, Chen YX , Zhang Y, Pi SY, Qu LC, Yu SB, Sui HJ. 2019. Existence and features of the myodural bridge in Gallus domesticus: indication of its important physiological function.－Anat Sci Int. 94(2):184-191. doi: 10.1007/s12565-018-00470-2.

4.Line 209: in what ways? more specific hypotheses based on some documented relevant biological aspects of penguins would be useful here.

At present, we distinguish differences, mainly on the basis of gross anatomy. For the next stage, related functional experiments will be carried out on the morphological abnormalities of different birds.

Page 12

1. formatting issues

Line 220: Rock pigeons

Page 17

1. formatting issues

Line 330: *Nephocaena phocaenoides*

Page 18

1. formatting issues

Line 340: *Trachemys scripta elegans*

2. formatting issues

Line 343: *Columbia livia*

3. formatting issues

Line 347: *Gallus domesticus*

4. formatting issues

Line 358: *Pygoscelis*

Page 19

1. formatting issues

Line 362: *Pygoscelis papua*

2.formatting issues

Line 364: *Pygoscelis papua*

3.The resolution of the photographs within the figures is currently not adequate, and many of the labels are not legible.

Thank you for your advice. The resolution and label of the image have been modified.

I have uploaded the image file to the "Pre-flight Analysis and Transformation Engine (PACE) Digital Diagnostic Tool" for inspection. I have deepened the color of the image.

Reviewer #2:

1.In the abstract, cut the sentence "The present authors suggest..." as it does not add to the main effort of this submission. Also, move the sentence "While foraging..." to follow the statement about the Gentoo penguins being the fastest diving birds.

Page 2

Line 19: ....interspaces. ~~The present authors suggest that the MDB has important physiological functions in humans.~~ The existence of the MDB....

Line 22: ....660 feet. While foraging, this penguin is able to reach speeds of up to 22 miles per hour. Gentoo penguins are also....

2.In the introduction, there are several myodural bridges described, so add the word "Each" at the beginning of the second sentence to make it, "Each myodural bridge (MDB) is..."

Page 3

Line 42: ....Each myodural bridge (MDB) is now described as a fibrous....

3.In the methods on "histological slices and staining" you use both "Picrosirius Red (PRS)" and "Picric acid-Sirius red" to describe the stain. While the two terms are synonymous, you might want to pick one version and use it consistently in this paper.

Page 6

Line 103: "Picric acid-Sirius red " was changed to "Picrosirius Red (PRS)" here and use it consistently in this paper.

....The results of Picrosirius Red staining were observed with a light microscope as well as a polarized light microscope.

4.In the results, the legends for Figures 3, 4, and 5 all have the same typo. I would suggest "In the atlanto-axial interspace, the dense fiber bundles (arrow) originating from the ventral SIDE OF THE RCDmi run ventrally" or something similar. I use the capital letters only for emphasis, they should be lower case in the revision.

Page 9

Line 159: ....the ventral side of the RCDmi run ventrally run ventrally....

Line 170: ....the ventral side of the RCDmi run ventrally run ventrally....

Line 181: ....the ventral side of the RCDmi run ventrally run ventrally....

5. In the Discussion, there should be a space between "In" and "1995" in the first sentence. You might want to define "natatores" as swimming birds since some readers (myself included) may not already be familiar with the term. Also, was that sentence meant to end with a question mark? That would make more sense in context. In the fourth paragraph, third sentence, change "found" to "exists". In the fifth paragraph, fourth sentence, change "ocean" to "depths".

Page 10

Line 188: In 1995 Hack et al. ....

Page 11

Line 194: "natatores" was changed to "main bird" .

....Therefore, what is unique about the penguin as a marine bird？

Page 12

Line 228: "found" was changed to "exists"

Page 13

Line 255: "ocean" was changed to "depths".
